# *miR-9-1* gene methylation and *DNMT3B* (rs2424913) polymorphism may contribute to periodontitis

**DOI:** 10.1590/1678-7757-2019-0583

**Published:** 2020-04-03

**Authors:** Marina Castro Coêlho, Ingrid Costa Queiroz, José Maria Chagas Viana, Sabrina Garcia de Aquino, Darlene Camati Persuhn, Naila Francis Paulo de Oliveira

**Affiliations:** 1 Universidade Federal da Paraíba Centro de Ciências da Saúde Programa de Pós Graduação em Odontologia João PessoaParaíba Brasil Universidade Federal da Paraíba, Centro de Ciências da Saúde, Programa de Pós Graduação em Odontologia, João Pessoa, Paraíba, Brasil.; 2 Universidade Federal da Paraíba Centro de Ciências da Saúde João PessoaParaíba Brasil Universidade Federal da Paraíba, Centro de Ciências da Saúde, João Pessoa, Paraíba, Brasil.; 3 Universidade Federal da Paraíba Centro de Ciências da Saúde Departamento de Odontologia Clínica e Social João PessoaParaíba Brasil Universidade Federal da Paraíba, Centro de Ciências da Saúde, Departamento de Odontologia Clínica e Social, João Pessoa, Paraíba, Brasil.; 4 Universidade Federal da Paraíba Centro de Ciências Exatas e da Natureza Departamento de Biologia Molecular João PessoaParaíba Brasil Universidade Federal da Paraíba, Centro de Ciências Exatas e da Natureza, Departamento de Biologia Molecular, João Pessoa, Paraíba, Brasil.

**Keywords:** Periodontitis, Epigenetic, DNA methylation, Polymorphism, Oxidative stress, MicroRNA

## Abstract

Genetic and epigenetic changes have been associated with periodontitis in various genes; however, little is known about genes involved in epigenetic mechanisms and in oxidative stress.

**Objective:**

This study aims to investigate the association of polymorphisms C677T in *MTHFR* (rs1801133) and −149C→T in *DNMT3B* (rs2424913), as well as the methylation profiles of *MTHFR, miR-9-1, miR-9-3, SOD1*, and *CAT* with periodontitis. The association between polymorphisms and DNA methylation profiles was also analyzed.

**Methodology:**

The population studied was composed of 100 nonsmokers of both sexes, divided into healthy and periodontitis groups. Genomic DNA was extracted from the epithelial buccal cells, which were collected through a mouthwash. Polymorphism analysis was performed through polymerase chain reaction-restriction fragment length polymorphism (PCR-RFLP), while methylation-specific PCR (MSP) or combined bisulfite restriction analysis techniques were applied for methylation analysis.

**Results:**

For *DNMT3B*, the T allele and the TT genotype were detected more frequently in the periodontitis group, as well as the methylated profile on the *miR-9-1* promoter region. There was also a tendency towards promoter region methylation on the *CAT* sequence of individuals with periodontal disease.

**Conclusion:**

The polymorphism −149C→T in *DNMT3B* (rs2424913) and the methylated profile of the *miR-9-1* promoter region are associated with periodontitis.

## Introduction

Periodontitis is defined as a chronic, inflammatory, and multifactorial disease; it is associated with a dysbiotic biofilm and characterized by the progressive destruction of the dental insertion apparatus.^1^ Epidemiological studies revealed that most adult individuals are affected by mild and moderate forms of this disease, while 5-20% of any population has severe periodontitis. In Brazil, the periodontitis prevalence is higher than the global average.^2^ If not treated, periodontitis is a risk factor for prominent noncommunicable diseases, including pneumonia, gastritis, diabetes mellitus, and chronic renal disorders; also, the number of lost teeth is predictive of mortality by cardiovascular conditions.^3^

As a multifactorial disease, genetic and environmental aspects are important factors that lead to the susceptibility to periodontitis. Environmental factors may alter the cellular epigenome, which may affect gene expression. The epigenome refers to chemical modifications that may occur on DNA and histones, and to the individual's non-coding RNA profile.^4^ Epigenetic marks comprise DNA methylation, chemical alterations on histones (methylation, acetylation, and phosphorylation), and small, non-coding RNA (microRNA).

DNA methylation occurs in the CpG dinucleotides, mainly in gene promoter regions, and controls expression at the transcriptional level by hindering the access of transcription factors to their binding sites. Methyl radicals are added to the DNA by DNA methyltransferases (DNMT), and these enzymes use the *S*-adenosyl methionine (SAM) radical as substrate. The SAM molecules are synthesized during the folate metabolic pathway by the methylenetetrahydrofolate reductase (MTHFR) activity. MicroRNAs, in turn, control the gene expression at the post-transcriptional level by biding to target messenger RNA (mRNA) and inhibiting their translation. Approximately 50% of the microRNA genes are associated with CpG islands, and their expression may be regulated by DNA methylation pathways.^5^

Genetic polymorphisms in *MTHFR* and *DNMT* may influence the DNA methylation profile; as mentioned, these enzymes originate the SAM radical and use it as substrate for DNA methylation, respectively. The polymorphism C677T *MTHFR* (rs1801133) reduces enzymatic activity, resulting in a decrease in SAM concentration. The polymorphism −149C→T *DNMT3B* (rs2424913), on the other hand, enhances the transcription of the sequence.^6^ In fact, some studies demonstrated that rs1801133 is associated with global and site-specific hypomethylation,^7^ while rs2424913 is associated with hypermethylation of specific genes.^8^

DNA methylation profile alterations have been described in periodontitis, especially in Toll-like receptor, cytokine, and metalloproteinase genes.^9^^,^^10^ Notably, some of these studies reported epigenetic alterations outside of gingival tissue, such as buccal epithelium and blood. This phenomenon may suggest these changes are not limited to the gingiva and, at the same time, reflect the cellular condition. Information on the methylation profile of microRNA genes (*miR*) and on the genes involved in the oxidative stress pathway are still scarce. However, the microRNA expression as well as antioxidant proteins, such as superoxide dismutase and catalase, are altered in periodontitis patients' gingival tissue cells and saliva.^11^^,^^12^

It speculated that genetic polymorphisms in DNA methylation pathway genes (*MTHFR* and *DNMT*) and methylation profile alterations in microRNA genes and in genes from the oxidative stress pathway may be involved in periodontitis pathogenesis. This study focused on these genes because, as mentioned earlier, *MTHFR* and *DNMT* are involved in DNA methylation processes and both polymorphisms and methylation of these genes are involved in inflammatory diseases,^13^^–^^15^ probably affecting the levels of products originated during the folate metabolic cycle, such as homocysteine and SAM radicals.^7^ Genes related to oxidative stress pathways, such as *SOD* and *CAT*, have been associated with periodontitis,^12^ while microRNA genes are involved in inflammatory diseases by silencing several mRNA targets related to cellular processes, including cellular homeostasis, proliferation, differentiation, development, growth, and apoptosis,^11^ particularly the miR-9 family targets transcripts of inflammatory cytokines.^16^^,^^17^ However, to the best of our knowledge, nothing is known, in the context of periodontitis, about genetic and epigenetic marks in the above genes.

Therefore, the main objective of this study was to investigate the association between the *MTHFR* rs1801133 and the *DNMT3B* rs2424913 polymorphisms, and methylation profiles from *MTHFR, miR-9-1, miR-9-3*, superoxide dismutase (*SOD1*), and catalase (*CAT*) genes with periodontitis. The study also assessed the association between these polymorphisms, the DNA methylation profiles, and periodontitis.

## Methodology

### Studied population and research ethics

The study was conducted according to the Declaration of Helsinki and was approved by the Research Ethics Committee (CAAE: 64578717.4.0000.5188). A convenience sample of unrelated nonsmoking subjects (30-70 years old) was recruited for this study from the patient pool of the Periodontics Clinic (Department of Clinical and Social Odontology) from May 2017 to November 2018. The studied population from the Northeast region of Brazil comprised 100 individuals of both sexes who were generally healthy; their diagnosis was based on the American Academy of Periodontology guidelines.^18^ The medical and dental history were recorded including the measurements of probing depth (PD), bleeding on probing, and clinical attachment level (CAL) at six sites *per* tooth. Following the diagnosis, the participants were divided into two groups according to the coauthor's supervision: the control group (n=50), which included individuals with no clinical signs of gingival inflammation, with CAL and PD ≤3 mm; and the chronic periodontitis group (n=50), comprising patients with at least 3 teeth presenting CAL and PD ≥5 mm and clinical signs of inflammation. Exclusion criteria for this study included smoking, diabetes, history of periodontal treatment, pregnancy, hepatitis, HIV infection, and use of anti-inflammatory medication in the last 6 months.

### Sample collection and DNA extraction

Buccal mucosa cells were collected from a one-minute mouthwash with 6 mL of autoclaved dextrose (3%), and after the collection 3 mL of TNE buffer was added. The samples were taken to the Laboratory of Molecular Genetics (Department of Molecular Biology), where nucleic acid extraction and molecular analysis were performed. Subsequently, the samples were centrifuged at 3,000 rpm for 10 min and the supernatant was discarded. Lysis solution was added to the pelleted epithelial cells and the samples were stored at −20ºC until DNA extraction. The genomic DNA was purified with 8 M ammonium acetate.^19^ The amount and the purity of the isolated DNA were measured with a Nanodrop spectrophotometer using the OD 260/280 ratio. The sample was considered pure when the mean value between two readings was 1.8 or greater.

### Genetic polymorphism analysis of *MTHFR* and *DNMT3B*

The analysis of the single nucleotide polymorphisms (SNPs) C677T *MTHFR* (rs1801133) and −149C→T *DNMT3B* (rs2424913) was performed through polymerase chain reaction–restriction fragment length polymorphism (PCR-RFLP), where DNA fragments are amplified by PCR and digested by a restriction enzyme. The presence or absence of the SNP was obtained according to the restriction enzyme activity. The 15-µL reactions contained 7.5 μL GoTaq^®^ G2 Hot Start Green Master Mix (Promega), 1 μL of each primer (10 μM), 1 μL of DNA, and nuclease-free water. PCR and enzymatic digestion conditions, as well as the chosen primers, were previously described.^15^^,^^16^^,^^20^^,^^21^ Genotypes were analyzed through vertical electrophoresis in 6% polyacrylamide gels, followed by coloring with silver nitrate or GelRed^®^ (Biotium). The 677 CC/CT/TT and the −149 CC/CT/TT genotypes were identified by their band pattern according to the literature.^15^^,^^16^^,^^20^^,^^21^

### Methylation analysis of *MTHFR, miR-9-1*, and *miR-9-3* gene promoters

Analysis of *MTHFR, miR-9-1*, and *miR-9-3* promoter methylation was performed using the methylation-specific PCR (MSP). To perform this technique, the purified DNA was transformed with sodium bisulfite and with hydroquinone for 3 h at 70°C for complete unmethylated cytosine conversion into uracil. The differences in the DNA sequences after the bisulfite treatment were detected by amplification with specific primers for methylated and non-methylated sequences, as previously described. The PCR conditions were also previously described.^22^^,^^23^ After amplification, the methylation profiles were visualized through vertical electrophoresis of 7 µl of amplified DNA in 6% polyacrylamide gels, followed by silver nitrate coloring. The profiles were categorized as methylated, with amplification for the methylated condition only; unmethylated, with amplification for the non-methylated condition; and partially methylated, when amplification was observed for both conditions.

### Methylation analysis of *SOD1* and *CAT* gene promoters

*SOD1* and *CAT* promoter methylation analysis was performed using combined bisulfite restriction analysis (COBRA), in which the DNA is submitted to a bisulfite treatment followed by a PCR amplification, and enzymatic digestion with restriction enzymes specific for transformed (C→U) or conserved CpG sites.^24^ The DNA was transformed as previously mentioned. The primer sequences for each gene (*CAT*: F:5'-GTTTTAATTGTTGAGTAATAAATGAGA-3' and R:5'-AAAAAAAACAACCTTCTTTTCA-3', 199 base pairs (bp); *SOD1*: F:5'-GGTTTTTTAATTGTTGGGTTAGAG-3' and R:5'- ACTCAACCAATCAACACCAC-3', 175 bp) were obtained using the Methyl Primer Express v1.0 (Applied Biosystems) software according to the promoter region sequence entered into the Genome Browser (chr21:31,659,622-31,668,931 and chr11:34,438,925-34,472,062, respectively). Fifteen µL reactions contained 7.5 µL GoTaq^®^ Hot Start Green Master Mix (1X; Promega), 2 µL of the primer sets (10 µM), 5 µL (∼100 ng) of transformed DNA, and nuclease-free water. Forty-cycle amplifications were performed for both sequences. For *CAT*, the annealing temperature was 55°C, while for *SOD* it was 51°C.

After amplification, 3 µL of the PCR products were digested by the restriction enzyme *Aci* I (GCGG; Thermo Fisher) for 3 h at 37°C, followed by vertical electrophoresis in a 6% polyacrylamide gel, and coloring with GelRed^®^. Methylation profiles were categorized as methylated or with enzymatic digestion when the GCGG sites were not transformed during treatment; unmethylated or without enzymatic digestion when the GCGG sites were transformed during the treatment; and partially methylated or with partial digestion. To ensure specificity of all methylation protocols, completely methylated and demethylated DNA controls were used (Cells-to-CpG™ Methylated & Unmethylated gDNA Control Kit, Life Technologies).

### Statistical analysis

All data were compiled in an Excel^®^ spreadsheet. Descriptive statistics was used for demographic data. The Hardy-Weinberg equilibrium (HWE) was calculated for each polymorphism using the chi-squared test. The Fisher's Exact or the chi-squared tests were also used for the analysis of possible associations between genotypic frequencies, allelic frequencies, methylation profiles, and the outcome (periodontitis), as well as the association between the methylation profiles and the presence of polymorphisms. All analyses were performed using the BioEstat 5.3 software. p-values <0.05 were considered statistically significant.

## Results

Demographic data are shown in Table 1.

**Table 1:** Demographic, polymorphism, and DNA methylation data

Demography	Control (n=50)	Periodontitis (n=50)	P value
Age (years)	38.7 (±7.5)	49.0 (±11.3)	0.0001
% men	12	22	0.001
% women	38	28	
Probing depth (mm)	≤ 3	≥ 5	
**Polymorphism**	**n= 50**	**n= 50**	
**DNMT3B genotype**			
CC	21 (42%)	12 (24%)	0.01*
CT	25 (50%)	24 (48%)	OR:4.47;
TT	04 (08%)	14 (28%)	[1.35;14.75]
Allele frequency %			
C	67	48	0.01*
T	33	52	OR: 2.19;
**MTHFR genotype**			
CC	26 (52%)	21 (42%)	
CT	18 (36%)	26 (52%)	>0.05
TT	06 (12%)	03 (06%)	
Allelle frequency %			
C	70	68	>0.05
T	30	32	
**DNA methylation**			
**MTHFR**	**n=40**	**n=40**	
M	01 (2.5%)	0 (0%)	
U	37 (92.5%)	36 (90%)	>0.05
M and U	02 (5%)	04 (10%)	
**miR-9-1**	**n= 40**	**n=40**	
M and U	31 (77.5%)	40 (100%)	0.0024#
U	09 (22.5%)	0	RR:1.29; [1.09;1.52]
**miR-9-3**			
M and U	40 (100%)	40 (100%)	>0.05
U	0	0	
**SOD1**	**n= 40**	**n= 38**	
M	0	0	
U	34 (85%)	30 (79%)	>0.05
M and U	06 (15%)	08 (21%)	
**CAT**	**n =40**	**n=40**	
M	01 (2.5%)	02 (5%)	
U	23 (57.5%)	18 (45%)	0.077#
M and U	16 (40%)	20 (50%)	

M= methylated, U= unmethylated, M and U= partially methylated

* Fisher's exact, #χ2 Test.

### Genetic polymorphism analysis

**Figure 1:**
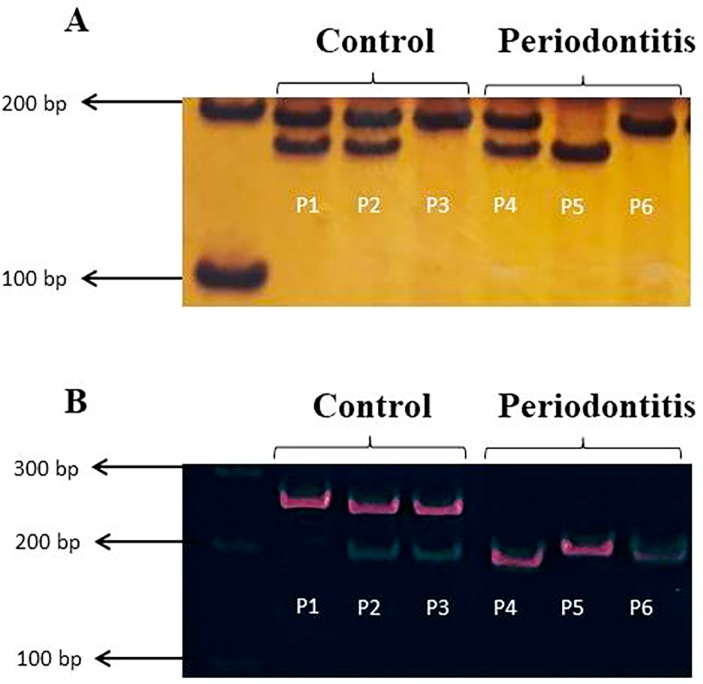
Representative bands of *MTHFR* and *DNMT3B* stained with silver nitrate or GelRed^®^ following the PCR-RFLP method for both control and periodontitis groups. (A) Amplicons for the *MTHFR* SNP in exon: CC=198 bp, CT=198, 175 and 23 bp (not shown), TT=175 and 23 bp (not shown); (B) Amplicons for the *DNMT3B* SNP in gene promoter: CC=230 bp, CT=230, 172 and 58 bp (not shown), TT=172 and 58 bp (not shown). P1-P6=patient 1–patient 6

Table 1 shows the genotypic and allelic distributions in the population studied. Both groups are in accordance with the HWE for each of the polymorphisms studied (*DNMT3B* (rs2424913): control, p=0.35 and periodontitis, p=0.78; *MTHFR* (rs1801133): control, p=0.31 and periodontitis, p=0.16). For *DNMT3B*, the T allele was more frequent in the periodontitis (52%) compared with the control group (33%; p=0.01). The same trend was observed for the TT genotype, which was more frequent in the periodontitis (28%) compared with the control group (8%; p=0.01). Individuals with the TT genotype are approximately four times more likely to develop periodontitis [OR: 4.47 (1.35;14.75)]. For *MTHFR*, the C allele and the genotypes CC and CT were the most common in both groups; therefore, no significant differences were observed between the groups (Table 1 and Figure 1).

### Methylation profile analysis

**Figure 2:**
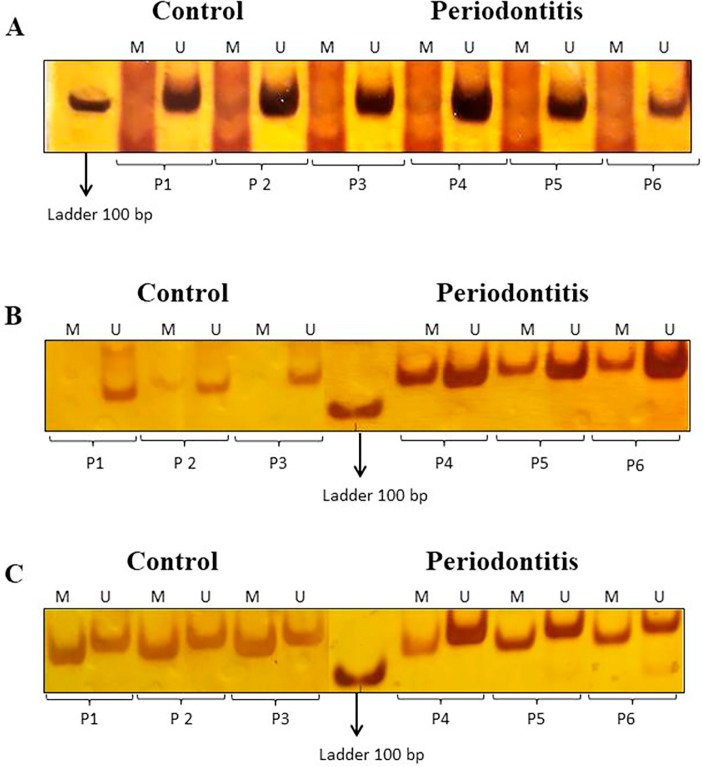
Representative bands of *MTHFR* and *miR-9* stained with silver nitrate following amplification by MSP method for both control and periodontitis groups. (A) Amplicons for methylated and unmethylated (198 bp) status of the *MTHFR* gene promoter; (B) Amplicons for methylated and unmethylated (110 bp) status of the *miR-9-1* gene promoter; (C) Amplicons for methylated and unmethylated (116 bp) status of the *miR-9-3* gene promoter. M=methylated, U=unmethylated. P1-P6=patient 1–patient 6.

**Figure 3:**
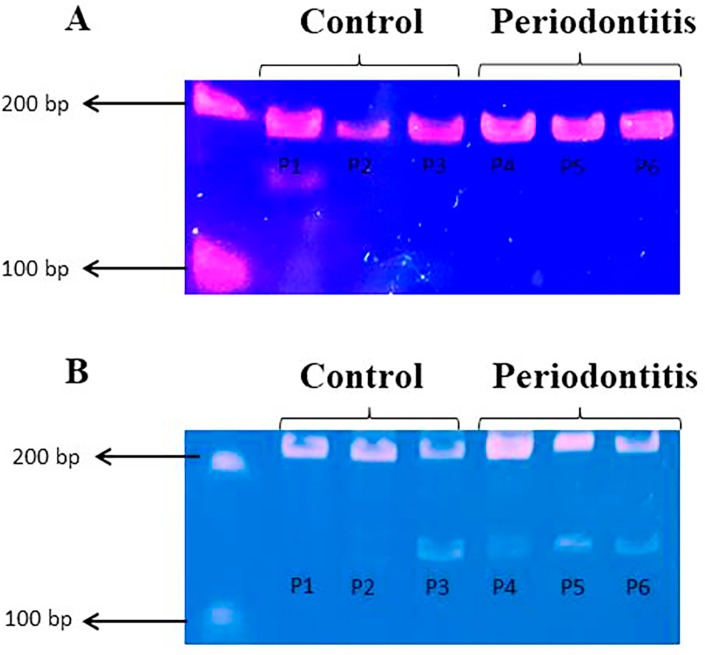
Representative bands stained with Gel Red of *SOD1* and *CAT* following the COBRA method for both control and periodontitis groups. (A) Amplicons after digestion for methylated (139 bp) and unmethylated (175 bp) status of the *SOD1* gene promoter; (B) Amplicons after digestion for methylated (167 bp) and unmethylated (199 bp) status of the *CAT* gene promoter. P1-P6=patient 1–patient 6.

For *MTHFR*, the unmethylated profile appeared more frequently in both groups (92.5% in the control group and 90% in the periodontitis group). For *miR-9-1*, methylation occurred in 100% of the periodontitis patients but only in 77.5% of the control group participants (p=0.0024). On the other hand, *miR-9-3* showed a partially methylated profile in both groups (100%; Table 1 and Figure 2). For *SOD1*, the unmethylated profile was the most frequent in both groups (85% of the control group and 79% of the periodontitis group). For *CAT*, although no significant difference was observed between the two groups, there was a tendency towards methylation in the periodontitis group, since the methylated profile was more common amongst these patients (55%=50% partially methylated +5% methylated) when compared with the control group (42.5%=40% partially methylated +2.5% methylated; p=0.077; Table 1 and Figure 3). The *DNMT3B* TT genotype and the *miR-9-1* methylation profile were considered for statistical association analysis, because both genes showed a statistically significant difference between the two groups. However, no association between these variables and periodontitis was found.

## Discussion

Periodontitis has been associated with genetic polymorphisms in several cellular pathways.^25^ From 2009 onwards,^9^ methylation analysis studies in the context of periodontal disease have markedly increased; however, many genes still need to be analyzed. This study focused on the genes that had not been previously studied in polymorphism or in methylation contexts, and are involved in DNA methylation (*MTHFR* and *DNMT3B*), oxidative stress (*SOD1* and *CAT*), and translation inhibition (non-coding *miR-9-1* and *miR-9-3*).

The population studied showed the TT genotype and the T allele for *DNMT3B* was more frequent in individuals with periodontitis. This gene is part of the DNA methyltransferase gene family, which acts by transferring the methyl radical (CH_3_) to DNA molecules with no previous methylation marks (*de novo* methylation). The C→T exchange in the promoter region enhances its own transcription and possibly causes a predisposition to abnormal de novo methylation and to transcription inhibition.^26^ Based on this possibility, the TT genotype could lead to hypermethylation and further silencing of important genes related to the protection against periodontitis. The homozygous TT genotype for the *DNMT3B* rs2424913 had already been previously associated with head and neck squamous cell carcinoma,^21^^,^^27^ while the CT genotype was associated with oral lichen planus and with an increase in expression of the protein encoded by this genotype.^15^ *DNMT3B* has been minimally explored in the periodontitis context and a recent study demonstrated that its expression increases after the administration of *Porphyromonas gingivalis* in experimental periodontitis.^28^

The *MTHFR* rs1801133 polymorphism showed no association with periodontitis. The C allele and the genotypes CC and CT were the most frequent in the entire population studied. The T allele and the TT genotype have been associated with other oral disease contexts, including lichen planus and squamous cell carcinoma.^29^^,^^30^ The enzyme encoded by this gene participates in the folate metabolism pathway, which produces the SAM radicals used by the DNMTs as methyl donors for DNA methylation. The C→T exchange reduces enzymatic activity and possibly leads to DNA hypomethylation.

In this study, the unmethylated *MTHFR* profile was recurrent among all participants. A previous study on *MTHFR* methylation in the buccal mucosa also identified the unmethylated profile as the most frequent amongst both young and elderly individuals.^31^ These data collectively demonstrate that this profile is common in buccal epithelial cells and is not associated with oral inflammation. The same CpG site analyzed herein has been studied by our group in patients with diabetic complications; the hypermethylated profile was detected in blood.^14^

The data of this study concerning promoter methylation in microRNA genes revealed that the partially methylated *miR-9-3* condition was the standard profile for buccal epithelial cells in individuals with or without periodontitis, while the *miR-9-1* methylation was more common in periodontitis patients. According to definitions found in a microRNA database (http://www.mirdb.org), all three loci for *miR-9* genes (1, 2 and 3) in the human genome can be transcribed into the same mature *miR-9* sequence.

The miRDB database shows *miR-9* may have 991 predictive targets. Hypermethylation of these genes and a decrease in their own expression may increase the levels of such targets, as has been recently shown by Marinho, et al.^32^ (2019). In this study, levels of the rap guanine nucleotide exchange factor, a *miR-9* target, were higher in the periodontitis patients' gingival crevicular fluid. The signaling cascade in which this factor participates may be involved in apoptosis, integrin-mediated signal transduction, and cellular transformation. Another target, the transforming growth factor β-1 (TGF-β1) receptor, is also expressed in the gingival tissue of individuals with periodontal disease.^16^ It binds to the anti-inflammatory cytokine TGF-β1, which is involved in tissue regeneration and cellular processes such as proliferation, growth, and differentiation.

*miR-9-1* hypermethylation has also been observed in cervical and ovarian cancer, and myeloma.^17^^,^^33^^,^^34^ A study with HeLa cells demonstrated that *miR-9* hypermethylation reduces its expression and is associated with enhanced interleukin 6 (IL-6) levels, a finding that may indicate that *IL-6* transcripts are targets for *miR-9*^17^. Interleukin 6 levels are higher in periodontitis patients when compared with those of control groups.^35^

The partially methylated *miR-9-3* condition, in which not all cells present methylation marks in the same CpG sites, had been previously observed for epithelial buccal cells of nonsmoker individuals, reassuring that partial methylation is the common profile for these cells.^22^

Regarding *CAT* and *SOD1* genes, CpG sites were chosen due to their location, because they are immersed in a CpG island near the gene promoter and act as recognition sites for restriction enzymes used in the COBRA technique. Sites −47 for *CAT* and −184 for *SOD1* have affinity for the Sp1 transcription factor, which positively controls transcription for these genes. This feature may suggest that both sites contribute to regulating each sequence's expression.^36^^,^^37^

Catalase and superoxide dismutase, the enzymes that are encoded by *CAT* and *SOD1*, respectively, are part of the human enzymatic antioxidant defense group that protects against oxidative damage derived from the reactive oxygen species (ROS). Thus, these enzymes modulate the extension of inflammatory reactions. Both enzyme levels have been evaluated in various contexts and many studies demonstrated a strong association between oxidative stress and periodontal disease. Most researches in this field evaluated transcript levels, protein expression, antioxidant enzymatic activity, total antioxidant capacity, and ROS levels.^12^ Nevertheless, methylation profile studies are scarce, and nothing was known about periodontitis patients.

For *SOD1*, the unmethylated profile was the most frequent for CpG site −184 in the entire studied population, indicating that this profile is standard for buccal epithelial cells. Similar to this study, an unmethylated profile for various *SOD1* promoter region CpG sites occurs in the cerebral cortex and blood cells in lateral amyotrophic sclerosis patients, as well as in the control individuals.^38^ Although the *SOD1* is generally considered to be a maintenance gene due to its ubiquitous and abundant expression, this induction of sequence is finely controlled by complex intracellular events. Alterations in the *SOD1* expression have been linked to periodontitis, however, data are still inconclusive, since some studies report increase in the SOD expression/activity while others indicate decline.^12^^,^^39^ Considering that gene expression may be controlled by DNA methylation, it is important to consider methylation studies on *SOD1* and periodontitis. If the analysis of this study had involved inflammation sites, such as SOD expression studies, some associations could have been detected. In any case, this study infers that the unmethylated profile at site −184 in *SOD1* is common for buccal epithelial cells and is not associated with periodontal inflammation.

For CpG site −47 in *CAT*, there was no significant difference between the groups, but the periodontitis group profile showed a tendency towards methylation. It has been demonstrated that methylated CpG sites near the *CAT* promoter region are associated with catalase transcription and expression decline in liver tumor cell lines and tumor tissues.^40^ The adverse effects of the *CAT* promoter methylation upon gene expression have also been reported in studies with hepatic cell lines treated with CuO nanoparticles.^40^

Catalase level reductions were detected in saliva of individuals with periodontitis when compared to healthy individuals^41^. This decline may be associated with gene methylation, which was a trend in the periodontitis group in this study. Additionally, the *CAT* hypermethylation observed by Punj, et al.^42^ (2017) occurred via ROS. Periodontal pathogens activate the host's defenses towards ROS formation, which in turn, contributes to the biofilm microorganisms' death. However, prolonged exposure to the ROS may induce a wide range of pathological reactions in the host's tissue, such as membrane lysis, nuclear fragmentation, and enzymatic activation or inactivation. These changes are likely to play a role in the pathogenesis of many inflammatory diseases, from periodontitis to systemic disorders.^42^ Thus, the trend towards methylation demonstrated in this study possibly derives from ROS release of buccal mucosa cells in response to periodontal pathogens. This tendency should be investigated in gingival tissue in addition to ROS and catalase levels analysis.

This study limitations consist of a low sample size for polymorphism studies due to a narrow sample collection period and to well-defined inclusion criteria, and for these reasons, data on the periodontitis group was not subgrouped by disease severity. However, the data of the study referred to the *DNMT3B* polymorphism rs2424913, which had not been previously studied in the periodontitis context. The finding of this study should be further explored in larger populations with different ethnic backgrounds before any conclusions can be drawn about the role of this gene in periodontitis. Although this study on DNA methylation did not involve the inflammation site, it still showed that profile alterations in buccal mucosa cells, especially in *miR-9-1*, may be involved in periodontitis.

As a multifactorial disease, periodontitis should be investigated focusing on an individual's genetic and epigenetic aspects. The latter can be modulated by individual habits and even be considered as therapeutic targets. Therefore, the data of this study contribute to the elucidation of molecular mechanisms involved in periodontitis development. Aside from cytokine genes, Toll-like receptors, matrix metalloproteinases, and other molecules mentioned in the literature, it demonstrated that genes involved in DNA methylation and translation inhibition may be involved in periodontitis. Molecular data can be collectively tabulated into a panel of genes indicating susceptibility to periodontal disease and be used as a tool for personalized medicine, contributing to better diagnosis, prognosis, and treatment.

In summary, the conclusion is that the genotype TT *DNMT3B* (rs2424913) and the methylated profile of the *miR-9-1* promoter region are associated with periodontitis. The *miR-9-1* promoter methylation yields a relative risk of 1.29 for periodontitis development, while a promoter region polymorphism in the *DNMT3B* causes individuals to be even 4 times more susceptible to periodontal disease.
